# Impact of Novel Guidelines on Multifactorial Control and Its Association with Mortality in Adult Patients with Hypertension and Newly Diagnosed Type 2 Diabetes: A 4-Year Prospective Multicenter Study

**DOI:** 10.1155/2021/9977840

**Published:** 2021-09-28

**Authors:** Ngoc-Thanh-Van Nguyen, Hoa Ngoc Chau, Nam Hoai Le, Hai Hoang Nguyen, Hoai-An Nguyen

**Affiliations:** ^1^Division of Cardiology, Department of Internal Medicine, Faculty of Medicine, University of Medicine and Pharmacy at Ho Chi Minh City, Ho Chi Minh City 700 000, Vietnam; ^2^Cardiology Department, Nhan Dan Gia Dinh Hospital, Ho Chi Minh City 700 000, Vietnam; ^3^Outpatient Department, University Medical Center, Ho Chi Minh City 700 000, Vietnam; ^4^University of Medicine and Pharmacy at Ho Chi Minh City, Ho Chi Minh City 700 000, Vietnam

## Abstract

**Methods:**

This prospective, observational study involved adult hypertensive patients with newly diagnosed type 2 diabetes mellitus at two university hospitals in Vietnam. The median time of follow-up was 4 years (August 2016–August 2020). The primary outcome was time to all-cause mortality.

**Results:**

246 patients were included with a mean age of 64.5 ± 10.4. 58.5% were females. 64.2% were categorized as high risk. At baseline, ischemic heart disease, dyslipidemia, and chronic kidney disease (CKD) were present in 54.9%, 67.1%, and 41.1% of patients. Renin–angiotensin–aldosterone inhibitor, metformin, and statin were prescribed in 89.8%, 66.3%, and 67.1%. Among three risk factors, LDL-c control was the hardest to achieve, increasing from 5.7% to 8.5%. In contrast, blood pressure control decreased from 56.1% in 2016 to 30.2% in 2020, when the second wave of COVID-19 hit our nation. While contemporary targets resulted in persistently low simultaneous control at 1.2%, significant improvement was observed with conventional criteria (blood pressure  < 140/90 mmHg, HbA1c < 7%, LDL-c < 70 mg/dl), increasing from 14.6% to 33.7%. During follow-up, the mortality rate was 24.4 events per 1000 patient-years, exclusively in patients with early newly diagnosed diabetes. Improving control overtime, not at baseline, was associated with less mortality. Conversely, age >75 years (HR = 2.6) and CKD (HR = 4.9) were associated with increased mortality.

**Conclusion:**

These findings demonstrated real-world difficulties in managing hypertension and newly diagnosed diabetes, especially with stringent criteria from novel guidelines. High-risk profile, high mortality, and poor simultaneous control warrant more aggressive cardiorenal protection, focusing more on aging CKD patients with early newly diagnosed diabetes.

## 1. Introduction

Hypertension is the leading preventable cause of premature mortality worldwide, affecting 31.1% of the world population [1]. In 2015, about 10 million deaths and 10 million ischemic events were attributed to hypertension [2]. Persistent disparity in hypertension prevalence and control was observed among different socioeconomic sectors. 75% of hypertensive patients reside in low- to middle-income countries, with a control rate of 10.1% [3, 4]. Better control was reported in high-income nations, reaching up to 70% in best-performing countries, yet the overall hypertension-related mortality rocketed overtime [5]. A contemporary approach focuses on risk stratification and comorbidity management to prevent and reverse hypertension-mediated organ damage.

Among coexistent conditions, diabetes emerges as significant comorbidity, with expanding epidemiology and accelerating cardiovascular mortality. Recent evidence has revealed the copredictive nature of hypertension and diabetes, further increasing the risk of cardiovascular events [6]. In patients with preexisting hypertension, the timing of diabetes diagnosis was associated with different mortality: the earlier the diagnosis, the higher the mortality [7].

Regardless of the order of diagnosis, risk factor control remains the cornerstone of treatment for coprevalent hypertension and diabetes. Simultaneous control together with lifestyle modification significantly reduced and delayed time to first cardiovascular event [8, 9]. Yet, a considerable gap exists between guideline and practice, as the rate of achieving and maintaining simultaneous control reported in the real-word study remains low [8, 10]. Specifically, the stringent, risk-based targets proposed by the state-of-the-art guidelines on hypertension and dyslipidemia predate failure to obtain simultaneous control, especially in poor-resource settings [11–13].

In Southeast Asia, coprevalence of diabetes and hypertension has been rising, ranging from 20.6% in India to 78.4% in Thailand [14]. In Vietnam, the first two decades of the twentieth century have witnessed a dramatic surge in the prevalence of hypertension and diabetes. Within 7 years, hypertension prevalence nearly doubled from 25.1% in 2008 to 47.3% in 2015. A similar trend was noticed for diabetes mellitus, especially in metropolitan areas, with a threefold increase from 3.8% in 2004 to 11.7% in 2016 [15–18]. Most studies were either limited to a single center or focused on one-time multiple risk factor control with short-term cardiovascular events. Therefore, we conducted the first multicenter prospective observational study to track multiple risk factor control over time, using present-day recommendations. We also examined the association between multifactorial control and mortality in this high-risk population.

## 2. Materials and Methods

### 2.1. Study Population

We designed a prospective, observational study, which was conducted at outpatient departments in the University Medical Center and Nhan Dan Gia Dinh hospital. The study was approved by the Ethics Committee of Biomedical Research at the University of Medicine and Pharmacy at Ho Chi Minh city and appropriate bodies at recruitment sites. All patients gave informed consent prior to participation. The study adhered to principles laid down in the Helsinki Declaration.

Inclusion criteria:
  1. Hypertensive patients with new-onset type 2 diabetes (diagnosed at least 2 years after hypertension onset)
  2. Age ≥18 years
  3. Regular follow-up in the previous 6 months
Exclusion criteria:
  1. Acute coronary syndrome, stroke, or hospitalization within 3 months
  2. Untraceable pharmacological/herbal treatment from out-of-recruitment sites
  3. Secondary hypertension
  4. Pregnancy or lactation
  5. Renal replacement therapy or eGFR<15 ml/min/1.73 m^2^
  6. Hepatic failure
  7. Concurrent participation in any interventional trial

### 2.2. Study Procedure

**Figure 1:**
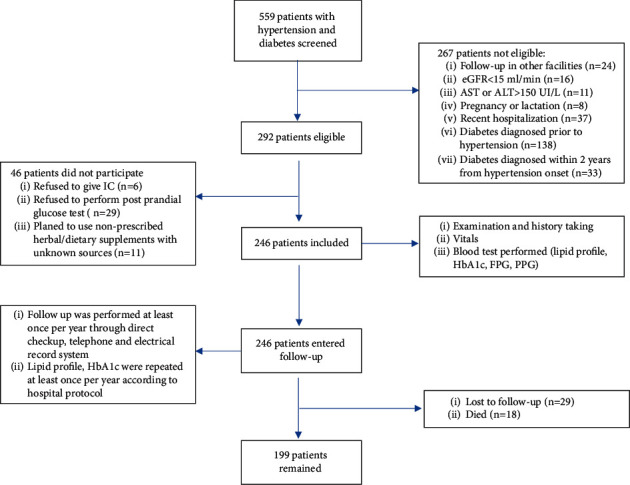
Flow diagram of the study procedure and patient selection. eGFR: estimated Glomerular Filtration Rate; ALT: alanine transaminase; AST: aspartate transaminase; IC: informed consent; PFG: fasting plasma glucose; PPG: postprandial plasma glucose; HbA1c: glycated hemoglobin.

Details on study procedure and patient selection are depicted in Figure 1.

### 2.3. Study Measures and Outcomes

Hypertension (HTN) was defined as either of the following:

1. Office blood pressure ≥140/90 mmHg on at least 2 visits, 1 to 4 weeks apart
2. Office blood pressure ≥180/110 mmHg and high cardiovascular risk
3. 24-hour blood pressure monitoring (Holter BP) ≥130/80 mmHg, daytime (awake) Holter BP ≥135/85 mmHg, nighttime (asleep) Holter BP ≥120/70 mmHg
4. Previously diagnosed with hypertension and currently receiving antihypertensive treatment

Diabetes (DM) was diagnosed using the 2016 ADA guideline for diabetes or currently on antiglycemic therapy [19]. We only recruited patients with diabetes diagnosed at least 2 years from the diagnosis of hypertension. As prediabetes and undiagnosed diabetes often precede clinical diagnosis, the “2-year” time point was chosen to distinguish the preexisting diabetic patients from new-onset individuals [7]. Early newly diagnosed diabetes was defined as diabetes diagnosed from 2 to <10 years after hypertension. Late newly diagnosed diabetes was defined as diabetes diagnosed at least 10 years after hypertension [7]. Family history was documented if at least one first-degree relative was diagnosed with hypertension (or diabetes). Body mass index (BMI) was calculated by dividing weight (kg) by height square (m^2^). Categorization of BMI was based on Asian Pacific criteria: underweight (<18.5), normal (18.5–<23), overweight (23–<25), and obese (>25). Nonsmokers were those who never smoke. Comorbidities were identified from the electronic medical record or newly diagnosed. Established ischemic heart disease included a documented positive noninvasive test, angiogram, or prior revascularization. Chronic kidney disease (CKD) was defined using the definition of the 2012 KDIGO guideline [20]. Heart failure (HF) was diagnosed using the 2016 European Society of Cardiology guideline on heart failure [21]. Atrial fibrillation (AF) must be documented either from a Holter electrocardiogram (ECG), a 12-lead ECG, or a 30-second ECG strip. Chronic obstructive pulmonary disease (COPD) and asthma were diagnosed using pulmonary function testing. Follow-up was carried out either via regular check-up or telephone.

Novel criteria for risk factor control were consistent with the most updated 2018–2019 European guidelines, including the following [11, 12]:

(1) Therapeutic range of blood pressure:
  - 120–<130/70–<80 mmHg for patients without chronic kidney disease and aged between 18 and 65
  - 130–<140/70–<80 mmHg for patients with chronic kidney disease or ≥65 years old
(2) HbA1c <7.0%
(3) LDL-c<55 mg/dL for very high-risk patients and <70 mg/dl for high-risk individuals [11–13]. Risk stratification was identified according to the 2019 European Society of Cardiology guidelines on diabetes [13].

At enrolment, each patient underwent physical examination and blood tests to determine baseline values on blood pressure, HbA1c, and LDL-c levels. For blood pressure, at least 2 measurements were performed at each visit, with an interval of 1 to 2 minutes. An additional measurement would be required if the first two differed by more than 10 mmHg. The average value of the last two readings was recorded. Both arms must be measured in the first visit, the one with higher reading was chosen for subsequent follow-up. Prior to each measurement, patients were required to relax for at least 5 minutes and to empty their bladder. No smoking, exercise, or caffeine consumption was allowed in the 30 minutes preceding blood pressure recordings. Standard cuffs were placed at the level of the heart, in a seated position with back and arms supported. Larger or thinner cuffs would be employed for larger or thinner arms. Talking or leg-crossing during measurement was prohibited.

We then collected data on blood pressure, HbA1c, and LDL-c at least once per year, or more frequently in case of medication titration/initiation or change in patients' condition. For patients who were lost to follow-up or died during the study, the last documented dataset of blood pressure, HbA1c, and LDL-c would serve as “end-of-follow-up” values. For patients who completed the study, data on the final follow-up were collected in July–August, 2020.

Continuous variables with normal distribution were presented as mean ± standard deviation; otherwise, the median (interquartile range) would be reported. Categorical variables were presented as proportion. We used the *t*-test or Wilcoxon rank-sum test for continuous variables and Chi-square or Fisher exact test for dichotomous or categorical variables. The Kaplan–Meier survival plot and log-rank test were used to describe the univariate analysis of the time-dependent event. Multivariable Cox proportional hazard regression was employed to investigate factors associated with the primary outcome. All tests were 2-tailed. A *p* value <0.05 was considered statistically significant. We used *R* for analysis.

## 3. Results

**Figure 2:**
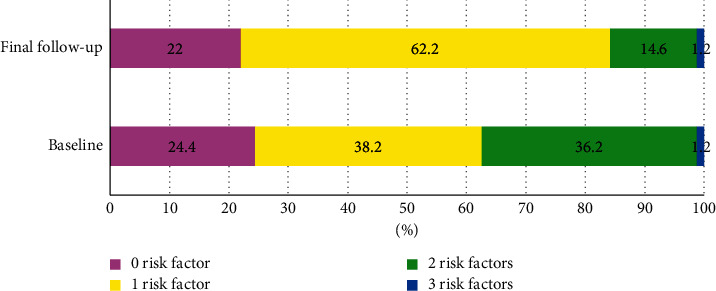
Change in the number of controlled risk factors overtime.

246 eligible hypertensive patients with newly diagnosed diabetes were recruited. Baseline characteristics on demographics, comorbidity, investigations, and medications are described in Table 1. The mean age was 64.5 years. 30.5% of patients were obese. The median time from hypertension to diabetes diagnosis was 4 years. 64.6% of patients were categorized as very high risk. At baseline, 54.9% had ischemic heart disease, and 41.1% had chronic kidney disease. 34.6% of patients had at least 2 comorbid conditions. The two most common antihypertensive medications were renin–angiotensin–aldosterone inhibitors (89.8%) and beta-blockers (72.0%). For antiglycemic management, the highest uptake was observed with metformin (66.3%) and Sulfonylureas (42.3%). Statin was noted in 67.1% of patients. During a 4-year follow-up, mortality occurred in 18 individuals (24.4 per 1000 patient-years) (Figure 2).

**Figure 3:**
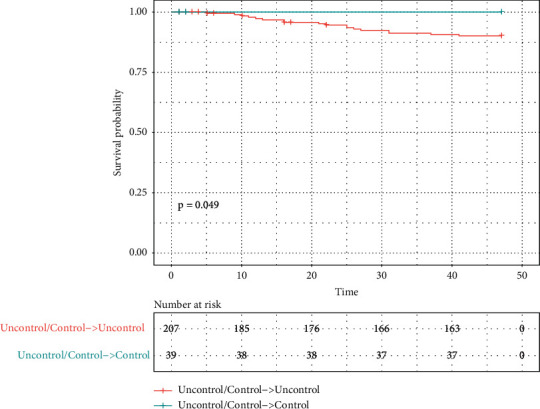
Kaplan–Meier curve for all-cause mortality in patients with and without two to three risk factors' control at final follow-up.

Table 2 demonstrates the control rate of individual and simultaneous risk factor according to the 2018-2019 guidelines from European Society of Cardiology. As there were a few cases of simultaneous control with risk-based targets at baseline (*n* = 3), at final follow-up (*n* = 3), and both (*n* = 0), we divided patients into 2 main categories: those who achieved at least 2 risk factors' control at final follow-up (*n* = 39) and those who did not (*n* = 207). Table 1 outlines their demographic, clinical, and treatment patterns. Table 2 demonstrates the control rate of individual and simultaneous risk factor according to the 2018-2019 guidelines from European Society of Cardiology.  Figure 3 depicts the Kaplan–Meier analysis for time to all-cause mortality between these two subgroups (*p* = 0.049).

**Figure 4:**
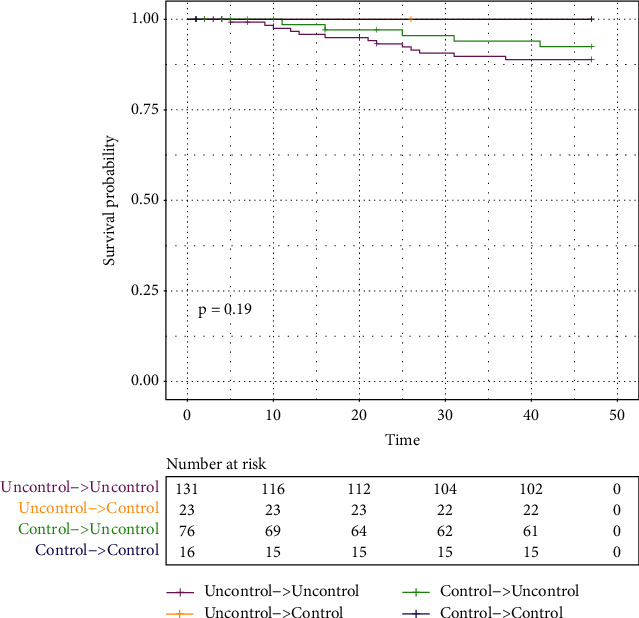
Changes in risk factor control and time to all-cause mortality in patients with hypertension and newly diagnosed diabetes.

Patients were further categorized into groups according to change of risk control overtime, including “control to control,” “uncontrol to control,” “control to uncontrol,” and “uncontrol to uncontrol.” No mortality was recorded in the “uncontrol/control to control” group, as opposed to the highest mortality in the “uncontrol to uncontrol” group (Figure 4). Specifically, all mortality occurred in the patients with early newly diagnosed diabetes.

**Figure 5:**
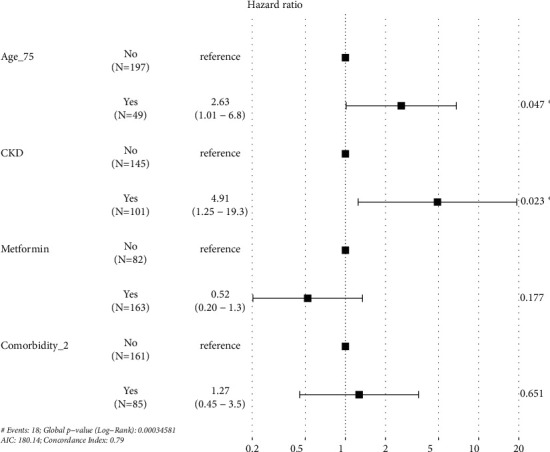
Multivariable Cox analysis of factors associated with all-cause mortality in patients with hypertension and newly diagnosed diabetes after 4 years.

**Figure 6:**
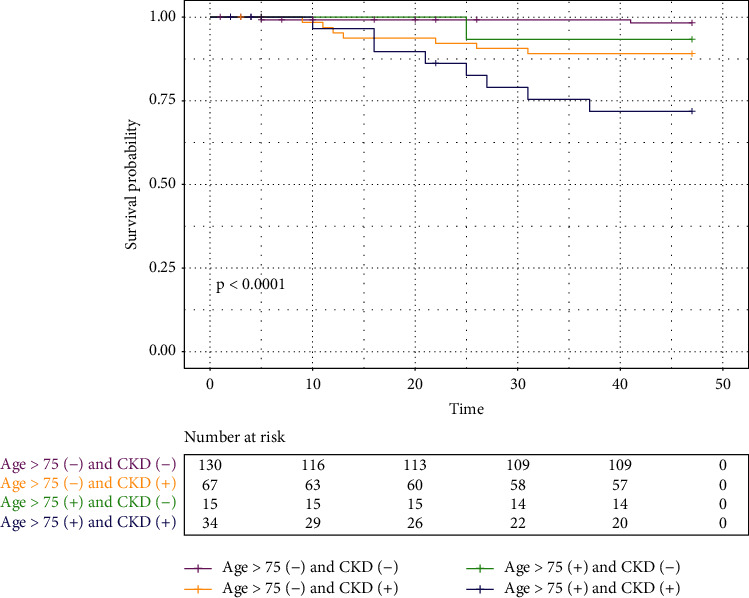
Kaplan–Meier for all-cause mortality in patients with and without CKD and age>75 or <75 years.

We performed univariate Cox progression on age, gender, BMI, smoking status, baseline risk, CAD, CKD, dyslipidemia, stroke, HF, AF, multimorbidity (≥ 2 comorbid conditions), uptake of RAAS inhibitor, statin, metformin, and ≥ 2 risk factors controlled at baseline. Statistical significance was achieved in age >75 years (HR = 4.3, 95% CI 1.7–11.0), baseline CKD (HR = 7.4, 95% CI 2.2–26.0), ≥2 comorbid conditions (HR = 3.0, 95% CI 1.2–7.8), and metformin (HR = 0.38, 95% CI 0.15–0.97) (Table 3). In multivariate Cox progression, only age>75 years (HR = 2.6, 95% CI 1.0–6.8) and CKD (HR = 4.9, 95%, CI 1.3–19.3) were associated with all-cause mortality (Figure 5). The Kaplan–Meier curve for time to all-cause mortality stratified by CKD and age>75 is plotted in Figure 6.

## 4. Discussion

Our study was the first multicenter, longitudinal research in Vietnam that tracked the changes of simultaneous risk factor control overtime using 2018–2019 guidelines of the European Society of Cardiology. Focusing on hypertensive patients with newly diagnosed diabetes, we described a predominantly female population (58.5%), who reported a high rate of CKD (41.1%), CAD (54.9%), and dyslipidemia (67.1%), together with high baseline uptake of RAAS inhibitor (89.8%), metformin (66.3%), and statin (67.1%).

Despite the high-to-very-high risk profile, the majority of patients only managed to control one to two risk factors (Figure 2). The low rate of LDL-c control was the main drive for poor simultaneous control (Table 2). This finding should be cautiously interpreted, taking into account the rigorous targets from novel guidelines (55 and 70 mg/dl) as opposed to more lenient classic thresholds (70 to 100 mg/dl), since slight modifications in criteria could lead to tremendous variations in control rate [10]. Compared to contemporary trials using the same cutoff, better LDL-c control was observed in our study (Table 4) [22, 23]. Likewise, we reported superior simultaneous control to present-day, large-scale studies when a similar set of criteria was employed (blood pressure<140/90 mmHg, HbA1c<7%, and LDL-c<70 mg/dl) (Table 4) [24, 25]. These facts infer the ubiquitous difficulty in achieving multifactorial control, which is further hindered by risk-based targets.

Different criteria lead to varying trends of simultaneous control. Conventional targets (blood pressure<140/90 mmHg, HbA1c<7%, and LDL-c< 70 mg/dl) resulted in an increasing pattern, from 14.6% to 33.7% whereas no change was observed with the state-of-the-art, risk-based recommendation. In regard to individual factor control overtime, blood pressure experienced a significant decrease in 2020, which may be associated with the second wave of COVID-19 in our nation. In July 2020, the first COVID-19 related mortality due to myocardial infarction was announced, followed by series of cardiovascular-related death, causing a significant surge in outpatient visits and increasing blood pressure readings. While the anxiety over COVID-19 could affect multiple risk factors, blood pressure was more prone to daily fluctuations.

In terms of mortality, our study demonstrated similar incidence (24.4 per 1000 patient-years) to contemporary trials in Western Asia (28.2 events per 1000 patient-years) and Europe (21.8 per 1000 patient-years) [5, 7]. Instead of baseline control, we put more emphasis on conversion to control at final follow-up. Prior real-world studies illustrated incremental effects of multifactorial control in reducing mortality in diabetes [8, 26]. However, these trials ignored the changing nature of control overtime. In our study, while the proportion of 3-factor control was extremely low, no mortality was observed in patients who achieved at least 2-factor control at final follow-up. This finding promotes continuous effort to achieve and maintain control.

In multivariable regression analysis, old age and CKD were associated with increased mortality. Escalating CKD incidence among diabetic patients, as well as increasing CKD-induced mortality merit careful attention [4, 27, 28]. Coexistent CKD was noted in 16.8% to 24.4% of the Asian diabetic population, whereas 43.8% of diabetes with microvascular complications reported eGFR below 60 ml/min [29–31]. Similarly, our high-risk patients exhibited a soaring prevalence of CKD (41.1%), which elaborated higher baseline prescription of RAAS inhibitor (89.8%) and statin (67.1%) compared to the Vietnamese subpopulation of the DiabCare study (46.8% and 40.3%, respectively) [32]. RAAS inhibitor and statin were shown to reduce cardiovascular events and slow down the rate of eGFR decline in CKD [29, 33, 34]. In the Nephropathy in Diabetes Type 2 trial (NID-2), intensified multifactorial control in patients with diabetic kidney disease significantly reduced mortality and major adverse cardiovascular events by 47% and 53%, respectively [35]. The protective effect was prominent early in the treatment course (3.84 years) and continued during 13 years of follow-up. Notably, the intensive arm in NID-2 trial utilized dual RAAS blockade, which is prohibited in current guidelines. Nevertheless, these observations uphold the practice of aggressive simultaneous control for better cardiorenal protection.

Aging was associated with increasing lifetime cumulative risk factors and decreasing functional capacity. In a meta-analysis of 19 elderly cohorts, frailty appeared as a strong predictor for medium- (RR = 9.49) and long-term mortality (RR = 7.94) [36]. Nonetheless, the assessment of frailty using standardized questionnaires was impractical in overburdened hospital facilities, necessitating another pragmatic approach, such as clinical phenotyping. In the REPOSI (REgistro POliterapi SIMI) registry, phenotypes with higher comorbidity burden and severe-to-total dependence were associated with increased mortality [37]. Likewise, decrement in quality of care and increment in hospitalization were observed with each additional comorbidity in elderly diabetic patients [38]. Our study demonstrated a higher proportion of multimorbidity (≥2 coexisting diseases) in patients >75 years (59.2%) as opposed to younger individuals (28.4%, *p* < 0.001). While the number of comorbid conditions alone was insufficient to fully expound adverse consequences in aging individuals with high cardiovascular risk [39], it did make a remarkable contribution to the worsening of overall health status. Therefore, the number of comorbidities could be considered as a simple indicator to forewarn physicians of imminently drastic deterioration in multimorbid elderly.

In our study, mortality occurred exclusively in patients with early newly diagnosed diabetes, supporting the hypothesis on mortality discrepancy among hypertensive patients with different timings of diabetes diagnosis. In early new-onset diabetes, insulin resistance acted as the common pathway, leading to the diagnosis of hypertension and diabetes within a short interval [6, 40]. The combined detrimental effects of hypertension and diabetes resulted in an excessive risk of mortality compared to late new-onset diabetes. In this subgroup, outcomes were mainly derived from long-term damage of hypertension.

Our findings imply three important clinical implications about the management of newly diagnosed diabetes in hypertensive patients. Firstly, a few patients achieved simultaneous control despite the high-risk profile. This finding was of particular concern as uncontrolled diabetes and hypertension were associated with increasing risks for severe COVID-19 complications [41–43]. Secondly, risk-based targets were hard to achieve and maintain in the long term. Improvement of control overtime, not at baseline, was associated with less mortality. Factors associated with improved control were lower baseline blood pressure, lipid, and HbA1c (Table 1). Altogether these facts suggest more aggressive control early in the course of treatment as well as continuous efforts to remain in control, especially for early newly diagnosed diabetes. Thirdly, a high index of suspicion is required for the elderly, frail individuals with multiple comorbidities. A proactive approach to detect and slow down CKD progression should be rigorously implemented, including efforts to reverse microalbuminuria and to optimize cardiorenal protection [26, 44, 45].

Our research should be interpreted in light of certain limitations. The study was designed as an observational, prospective study, with no randomization. We did not explore the effect of lifestyle modification or dosing of RAAS inhibitors. For risk factor control, we only compared baseline and final values and disregarded interval measurements, thereby overlooking possible fluctuations between follow-ups. Study recruitment sites could serve as a selection bias, as tertiary settings were often associated with more severe diseases and aggressive control. Self-reported variables were subject to recall bias, such as the history of smoking, family history, and duration of diseases. Finally, the study was initiated in 2016, when sodium-glucose cotransporter-2 inhibitor and glucagon-like peptide-1 receptor antagonist were not widely available in Vietnam. Therefore, the synergistic effect of modern cardiorenal protective therapy on high-risk diabetes could not be evaluated [44, 45]. More well-designed, long-term randomized trials should be carried out to better determine the trend in control rate, as well as its association with cardiovascular outcomes, specifically in the era mixed with a plethora of breakthroughs in metabolic management and urgent pandemic-induced challenges in healthcare delivery.

## 5. Conclusion

Our study depicted a high-risk population, who experienced high mortality incidence despite a high intake of RAASi and statin. Simultaneous control of blood pressure, HbA1c, and LDL-c was comparable to most contemporary studies, yet still far from expectation. Poor control rate, especially in regard to LDL-c, was further aggravated by rigorous targets from the state-of-the-art guidelines. Improving control over the course of treatment was associated with less mortality. Aggressive simultaneous control and integrative cardiorenal management should be enhanced, especially in aging CKD patients with early newly diagnosed diabetes.

## Figures and Tables

**Table 1:** Baseline patients' characteristics.

Variables	Overall (*n* = 246)	Uncontrol/control to control (*n* = 39)	Uncontrol/control to uncontrol (*n* = 207)	*p*
*Demographics*				
Age (year)	64.5 ± 10.4	62.3 ± 9.3	65.0 ± 10.6	0.147
Female (%)	58.5%	61.5%	58.0%	0.812
BMI (kg/m^2^)	23.7 (22.3, 25.6)	24.0 (22.9, 25.3)	23.6 (22.2, 25.7)	0.610
Smoking (%)	26%	20.5%	27.1%	0.512
Family history of HTN (%)	62.6%	56.4%	63.7%	0.490
Family history of DM (%)	26.0%	23.1%	26.6%	0.797
Systolic blood pressure (mmHg)	130 (130–140)	130 (120–130)	130 (130–140)	0.013^∗^
Diastolic blood pressure (mmHg)	80 (70–80)	80 (70–80)	80 (70–80)	0.238
Early new-onset diabetes (%)	89.4	84.6	90.3	0.434

*Comorbidity*				
CAD (%)	54.9%	48.7%	56.0%	0.504
CKD (%)	41.1%	33.3%	42.5%	0.373
AF (%)	6.5%	7.7%	6.3%	0.725
HF (%)	6.5%	2.5%	7.2%	0.480
Stroke (%)	4.9%	5.1%	4.8%	1
COPD/asthma (%)	0.4%	0%	0.5%	1
Cancer (%)	2.4%	0%	2.9%	0.594
≥2 comorbid conditions (%)	34.6%	25.6%	36.2%	0.274

*Investigations*				
HbA1c (%)	7.2 ± 1.2	6.8 ± 0.7	7.3 ± 1.2	0.004^∗^
Fasting plasma glucose (mg/dl)	134.9 ± 32.5	119.1 ± 21.4	137.9 ± 33.5	0.001^∗^
Postprandial plasma glucose (mg/dl)	172.7 ± 49.8	155.1 ± 31.4	176.0 ± 51.9	0.016^∗^
Cholesterol total (mmol/L)	4.9 ± 1.2	4.9 ± 1.3	4.9 ± 1.2	0.896
LDL-c (mmol/L)	3.1 ± 0.9	3.1 ± 1.1	3.2 ± 0.9	0.532
HDL-c (mmol/L)	1.1 ± 0.5	1.3 ± 0.8	1.1 ± 0.4	0.009^∗^
Triglyceride (mmol/L)	2.3 ± 1.2	1.8 ± 0.8	2.4 ± 1.3	0.013^∗^

*Medications*				
ACEi/ARB (%)	89.8	92.3	89.4	0.775
Beta-blocker (%)	72.0	61.5	73.9	0.166
Calcium channel blocker (%)	51.2	46.2	52.2	0.141
Diuretic (%)	17.5	12.8	18.4	0.545
Insulin (%)	3.7	0	4.3	0.362
Metformin (%)	66.3	79.5	64.3	0.092
Sulfonylurea (%)	42.3	30.8	44.4	0.159
DPP4-inhibitor (%)	6.5	7.7	6.3	0.725
Statin (%)	67.1	59.0	68.6	0.323

^∗^Statistically significant. Uncontrol: uncontrol at final follow-up; control: control at final follow-up; uncontrol/control: uncontrol or control at baseline; BMI: body mass index, HTN: hypertension; DM: diabetes mellitus; CAD: coronary artery disease; CKD: chronic kidney disease; AF: atrial fibrillation; HF: heart failure; COPD: chronic obstructive pulmonary disease, ACEi: angiotensin-converting enzyme inhibitor; ARB: angiotensin receptor blocker.

**Table 2:** Control rate of individual risk factor at baseline and final follow-up according to 2018-2019 guidelines on hypertension and diabetes from European Society of Cardiology.

	At baseline (%)	At 4 years (%)	*p*
Blood pressure control (%)	56.1	30.2	<0.0001^∗^
SBP control (%)	63.0	39.2	<0.0001^∗^
DBP control (%)	82.1	65.3	<0.0001^∗^
LDL-c control (%)	5.7	8.5	0.23
TG < 150 mg/dl (%)	32.9	36.8	0.38
HDL-c >40–50 mg/dl (%)	36.6	19.3	<0.0001^∗^
HbA1c control (%)	52.4	55.6	0.49
Simultaneous control (%)	1.2	1.2	1

^∗^: statistically significant. SBP: systolic blood pressure; DBP: diastolic blood pressure; TG: triglyceride; LDL-c: low-density lipoprotein-cholesterol; HDL-c: high-density lipoprotein-cholesterol; HbA1c: glycated hemoglobin.

**Table 3:** Univariate Cox analysis of factors associated with all-cause mortality in patients with hypertension and newly diagnosed diabetes after 4 years.

Variables	HR	95% CI	*p*
Age>75	4.3	1.7–11.0	0.002^∗^
Gender (male)	1.2	0.5–3.0	0.768
Smoking	0.43	0.1–1.9	0.256
BMI	0.68	0.2–2.1	0.488
Very high risk	0.77	0.3–2	0.585
CKD	7.4	2.2–26.0	0.001^∗^
CAD	0.97	0.38–2.5	0.994
Dyslipidemia	1.9	0.69–5.4	0.211
HF	3.1	0.71–13	0.135
AF	1.8	0.4–7.6	0.453
Stroke	1.2	0.16–9	0.865
≥2 comorbid conditions	3.0	1.2–7.9	0.022^∗^
ACEi/ARB	0.36	0.1–1.1	0.068
Statin	1.7	0.6–5.2	0.345
Metformin	0.38	0.15–0.97	0.042^∗^
≥2 risk factors controlled at baseline	0.79	0.3–2.1	0.63

^∗^Statistically significant. BMI: body mass index; CAD: coronary artery disease; CKD: chronic kidney disease; AF: atrial fibrillation; HF: heart failure; COPD: chronic obstructive pulmonary disease; ACEi: angiotensin converting enzyme inhibitor, ARB: angiotensin receptor blocker.

**Table 4:** Comparison of LDL-c and simultaneous control between our study and present-time trials according to different sets of criteria [22–25].

	Cutoff	Other study	Our study
Year		2019	2021
LDL-c control	<100 mg/dL	43.5%	62.2%^∗^
	<70 mg/dL	24.5%	27.2%^∗^

Multifactorial control	BP < 140/90 mmHg, HbA1c<7%, LDL-c<100 mg/dL	29.5%	33.7%^∗^
		21.6%	33.7%^∗^
	BP < 130/80 mmHg, HbA1c<7%, LDL-c<100 mg/dl	13.0%	23.3%^∗^

^∗^Control rate achieved in at final follow-up. LDL-c: low-density lipoprotein-cholesterol.

## Data Availability

The data that support the findings of this study could be partially available upon reasonable requests to hoachau@ump.edu.vn or vanntnguyen.md@ump.edu.vn. Strict restrictions apply to the availability of these data, for some are still under analysis and not yet published.
